# Systematic review and meta-analysis for the impact of rod materials and sizes in the surgical treatment of adolescent idiopathic scoliosis

**DOI:** 10.1007/s43390-022-00537-1

**Published:** 2022-06-23

**Authors:** Dawn Bowden, Annalisa Michielli, Michelle Merrill, Steven Will

**Affiliations:** DePuy Synthes Spine, Johnson & Johnson Medical Devices, 325 Paramount Drive, Raynham, MA 02767 USA

**Keywords:** Adolescent spine deformity, Surgery, Outcomes, Complications, Rods, Diameter, Material

## Abstract

**Purpose:**

To assess surgical and safety outcomes associated with different rod materials and diameters in adolescent idiopathic scoliosis (AIS) surgery.

**Methods:**

A systematic literature review and meta-analysis evaluated the surgical management of AIS patients using pedicle screw fixation systems (i.e., posterior rods and pedicle screws) with rods of different materials and sizes. Postoperative surgical outcomes (e.g., kyphosis and coronal correction) and complications (i.e., hyper/hypo-lumbar lordosis, proximal junctional kyphosis, revisions, reoperations, and infections) were assessed. Random-effects models (REMs) pooled data for outcomes reported in ≥ 2 studies.

**Results:**

Among 75 studies evaluating AIS surgery using pedicle screw fixation systems, 46 described rod materials and/or diameters. Two studies directly comparing titanium (Ti) and cobalt–chromium (CoCr) rods found that CoCr rods provided significantly better postoperative kyphosis angle correction vs. Ti rods during a shorter follow-up (0–3 months, MD = − 2.98°, 95% CI − 5.79 to − 0.17°, *p* = 0.04), and longer follow-up (≥ 24 months, MD = − 3.99°, 95% CI − 6.98 to − 1.00, *p* = 0.009). Surgical infection varied from 2% (95% CI 1.0–3.0%) for 5.5 mm rods to 4% (95% CI 2.0–7.0%) for 6 mm rods. Reoperation rates were lower with 5.5 mm rods 1% (95% CI 0.0–3.0%) vs. 6 mm rods [6% (95% CI 2.0–9.0%); *p* = 0.04]. Differences in coronal angle, lumbar lordosis, proximal junctional kyphosis, revisions, and infections did not differ significantly (*p* > 0.05) among rods of different materials or diameters.

**Conclusion:**

For AIS, CoCr rods provided better correction of thoracic kyphosis compared to Ti rods. Patients with 5.5 mm rods had fewer reoperations vs. 6.0 and 6.35 mm diameter rods.

**Level of evidence:**

III.

**Supplementary Information:**

The online version contains supplementary material available at 10.1007/s43390-022-00537-1.

## Introduction

Adolescent idiopathic scoliosis (AIS) is the most common spinal deformity among the pediatric population, occurring in patients aged 10–18 years. Its idiopathic nature necessitates that defined causes of scoliosis (i.e., vertebral or neuromuscular disorders, and other syndromes) have been ruled out. The worldwide prevalence of AIS ranges from 0.47 to 12% and varies according to genetics, age, and gender [1–9]. AIS more commonly affects girls than boys, with a female to male ratio of 3.1–1.5 [1]. Moreover, the risk of AIS in girls increases more than boys with increasing age [10]. A higher prevalence of AIS has been reported in the African-American population (9.7%) compared to the Caucasian population (8.1%) [1].

AIS treatment depends on the severity of the curvature [10–13]. The objectives of surgery in adolescents with significant and/or progressive curvature include achieving a solid fusion and arresting curve progression, achieving permanent deformity correction, improving functional outcomes, improving physical appearance, and suppressing the development of problems in adulthood (i.e., back pain, degenerative changes, functional impairment, and cardiopulmonary compromise). Additional desirable characteristics include preventing surgical complications (e.g., neurological injury, dural tears, position-related complications, gastrointestinal complications, infections and wound complications, implant-related issues, pseudoarthrosis, curve progression, adding-on, and proximal junctional kyphosis [14]) while preserving as many mobile spine segments as possible.

There are multiple factors which contribute to the successful correction of AIS and to minimizing the complications brought about by the surgical treatment. Spinal fixation rods play an important role in the outcomes of spinal deformity surgery as they impact the success of the restoration of global alignment and balance. Hence, surgeons require rods that deliver optimal alignment and meet the needs of each individual patient. A better understanding of the clinical performance of various types of rods available for AIS would help healthcare providers and payers prioritize resource allocation and develop more effective and targeted interventions for the surgical treatment of AIS. The objectives of this study were to assess current evidence for the surgical and safety outcomes associated with rod materials and dimensions for the operative treatment of AIS.

## Materials and methods

### Study design and approach

The systematic literature review and meta-analysis was conducted according to the Preferred Reporting Items for Systematic Reviews and Meta-Analyses (PRISMA) guidelines [15]. Electronic searching of MEDLINE, Embase, KOSMET: Cosmetic Science, APA PsycInfo, and BIOSIS Previews was carried out on November 10, 2020 using the search terms: (spine* OR vertebra*) AND (fusion AND stabilization) AND (rods) AND (child* OR pediatric OR adolescent*). English-language studies published on or after January 1, 2010 evaluating AIS surgical management (patient age 10–18 years) using pedicle screw fixation systems (i.e., posterior rods and pedicle screws), including, but not limited to, ponte osteotomy, revision surgeries, and primary or secondary surgeries, were eligible. The focus of the systematic literature review and meta-analysis was to summarize published clinical evidence from studies conducted in human patients. Biomechanical ex vivo studies, animal studies, and cadaver studies were not included in the evaluations.

### Outcome measures

Surgical outcomes included postoperative kyphosis and coronal correction. Postoperative complications included hyper/hypo-lumbar lordosis, proximal junctional kyphosis, revisions, reoperations, and infections.

### Study selection and quality assessment

**Fig. 1:**
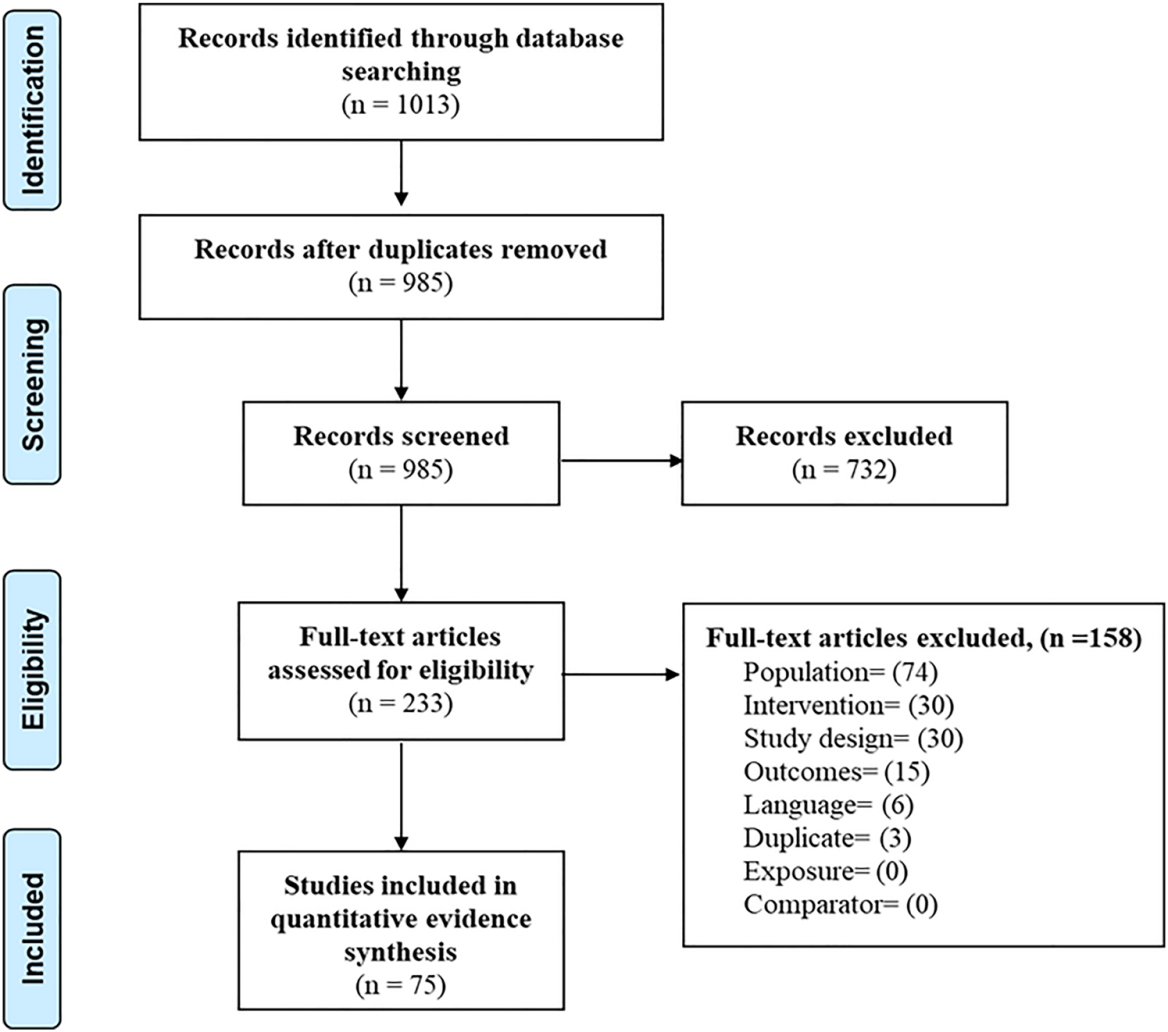
PRISMA flow diagram showing study selection

Two reviewers independently applied inclusion/exclusion criteria to screen de-duplicated titles and abstracts. Potentially relevant citations were checked in a full-text screening. Disagreements were resolved through discussion and reasons for exclusion were recorded (Fig. 1). Included studies were critically appraised and ranked as low/good/high-quality evidence using the Evidence level and Quality Guide from Johns Hopkins Nursing Evidence-Based Practice [16, 17].

### Evidence synthesis and statistical analysis

Qualitative and quantitative synthesis (using meta-analysis) were performed. Qualitative synthesis included summarizing individual studies and describing their results with respect to the relevant outcomes. Meta-analysis was performed for outcomes that were reported in ≥ 2 studies. For continuous outcome measures, inverse variance random effects models (REMs) estimated pooled mean differences (MDs). Pooled standardized mean differences (SMDs) were used for pain scores since the studies used different pain scales. Means and standard deviations (SDs) were extracted from individual studies or derived from medians with interquartile ranges or means with *p* values. For dichotomous outcomes, Mantel–Haenszel REMs estimated pooled risk ratios (RRs). For the pooled summary statistics for each outcome in the surgical and non-surgical intervention groups, inverse variance REMs were used. All effect sizes were reported with 95% confidence intervals (CI). The *χ*^2^ test was used to test for statistical heterogeneity (*α* = 0.05) and heterogeneity was quantitatively evaluated using *I*^2^ statistics. Statistical significance was set at *p* ≤ 0.05. RevMan version 5.4 was used for the evidence synthesis and statistical analysis.

## Results

### Study identification and selection

Among 75 studies meeting the inclusion criteria (Fig. 1), 46 described the rod material and diameter. Titanium alloy (Ti) rods were used in most studies (*n* = 32), followed by cobalt–chromium (CoCr; *n* = 16), and stainless steel (SS; *n* = 8). Rod diameter varied from 4.5 [18] to 6.5 mm [18]; however, the most common rod diameters were 5.5 mm [19, 20], 6.0 mm [21, 22], and 6.35 mm [23, 24]. Table 1 provides a description of the 75 included studies.

**Table 1:** Characteristics of studies (*n* = 75) that fulfilled the inclusion criteria for the systematic review and meta-analysis

Study	Study design	No. of patients	Gender		Patient Characteristics	Study groups (no. of patients)	Type of surgery	Mean age at surgery (years)	Follow-up Mean (SD), months)
			Male (*n*)	Female (*n*)					
Machino et al. (2020) [25]	Cohort study	67	8	59	AIS	Posterior rods and pedicle screws	PLIF	14.4 mean	NR
Kluck et al. (2020) [26]	Cohort study	99	NR	NR	AIS	Posterior rods and pedicle screws	NR	14 ± 2 years	NR
Shen et al. (2020) [27]	Cohort study	19	0	19	AIS	Posterior rods and pedicle screws	PLIF	15.6 ± 2.1	NR
Miyazaki et al. (2020) [28]	Cohort study	27	NR	NR	AIS	Hypokyphotic normal–hyperkyphotic	PSF with double-rod rotation	NR	NR
Feeley et al. (2019) [29]	Cohort study	31	8	23	AIS	Lenke A/B Lenke C	PLIF PLIF	NR	NR
Chang et al. (2019) [30]	Cohort study	28	NR	NR	AIS	LIV L3 LIV L4	PLIF PLIF	NR	NR
Violas et al. (2019) [31]	Case series	23	5	19	AIS	Posterior rods and pedicle screws	PLIF	14.75	37
Newton et al. (2018) [32]	Cohort study	134	40	94	AIS	Posterior rods and pedicle screws	PLIF	14.7 ± 2	NR
Lastikka et al. (2019) [33]	Cohort study	90	20	70	AIS	Circular rods Reinforced rods	PLIF PLIF	15.6 ± 2.1	NR
Mac-Thiong et al. (2019) [34]	Cohort study	80	10	70	AIS	Posterior rods and pedicle screws	PLIF	14.5 ± 2.2	NR
Uehara et al. (2019) [35]	Cohort study	69	4	65	AIS	Posterior rods and pedicle screws	PLIF	14.8 ± 2.5	NR
Zhang et al. (2018) [36]	Cohort study	36	10	26	AIS	Rod-link reducer (RLR) Traditional corrective techniques (TCT)	PLIF PLIF	NR	NR
Clément et al. (2019) [37]	Cohort study	111	NR	NR	AIS	Hypokyphosis Normokyphosis	PLIF PLIF	NR	NR
Miyazaki et al. (2019) [23]	Cohort study	24	3	22	AIS	Simultaneous double-rod rotation technique (SDRRT)" "Simultaneous double-rod rotation technique (SDRRT) + direct vertebral rotation (DVR)	PLIF PLIF	NR	NR
Ilharreborde et al. (2018) [38]	Case series	60	6	54	AIS	Posterior rods, pedicle screws, sublaminar bands, hooks	PLIF	15.4 ± 2	28.2 ± 4
Etemadifar et al. (2018) [19]	Randomized controlled trials (RCT)	59	22	37	AIS	CoCr–Ti rods Ti–Ti rods	PLIF PLIF	14.14 ± 1.41	NR
Sabah et al. (2018) [21]	Cohort study	63	27	54	AIS	CoCr rod Ti alloy TA6V rod (Ti)	PSF PSF	15 ± 2	42 ± 17
Sudo et al. (2018) [22]	Case series	39	0	39	AIS	Simultaneous double-rod rotation technique	NR	NR	48
Ketenci et al. (2018) [39]	Cohort study	83	NR	NR	AIS	AIS group—T2 group AIS group—T3 group AIS group—T4 group Control group	Posterior	15.1	NR
Kaliya-Perumal et al. (2018) [40]	Cohort study	88	10	78	AIS	Group 1: concave group Group 2: convex group	NR NR	14.1 ± 2.2	47.7 ± 14.6 49.5 ± 17.1
Faldini et al. (2018) [20]	Case series	36	4	32	AIS	Group A Group B Group C	NR NR NR	15.1 ± 1.8 years	24 (12–36)
Berger et al. (2018) [41]	Case series	30	5	25	AIS	Pedicle screw and rod system	Posterior	15	NR
Seki et al. (2018) [42]	Case series	40	3	37	AIS	Lenke Type I Lenke Type II Lenke Type III or IV	Posterior	14.1 ± 3.1	NR
Cheung et al. (2018) [43]	Randomized controlled trials (RCT)	23	6	17	AIS	CTA (control) SNT (intervention)	Posterior spinal exposure Posterior spinal exposure	15 ± 2.3	NR
Allia et al. (2018) [44]	Case series	68	60	8	AIS	Group D + Group D−	NR NR	NR NR	NR NR
Luo et al. (2017) [45]	Cohort study	57	12	45	AIS	"Group 1 (postop TK ≥ 20°)" Group 2 (postop TK < 20°)	Posterior Posterior	14.39 ± 1.82	NR
Zifang et al. (2017) [46]	Cohort study	81	14	67	AIS	Convex-rod derotation group Concave-rod derotation group	Posterior Posterior	15.0 ± 2.3 14.6 ± 2.2	NR
Ohrt-Nissen et al. (2017) [47]	Cohort study	139	22	117	AIS	Hybrid construct (HC) Standard construct (SC) Modified construct (MC)	Posterior midline approach Posterior midline approach Posterior midline approach	NR	NR
Faldini et al. (2017) [48]	Case series	30	4	26	AIS	Combined DVR and vertebral translation	Posterior approach	14.8	32.4
Lamerain et al. (2017) [49]	Cohort study	61	14	47	AIS	Group A: decreased thoracic lordosis Group B: normal (35–50 degrees) thoracic kyphosis	Posterior spinal fusion Posterior spinal fusion	15.4	37.4
Le et al. (2017a) [50]	Cohort study	42	5	37	AIS	CoCr SS Ti	Posterior approach Posterior approach Posterior approach	17	26.4
Chang et al. (2017) [51]	Cohort study	64	NR	NR	AIS	AL3 (flexible) BL3 (rigid)	Posterior surgery Posterior surgery	15 ± 1.9 (*p* = 0.856) 15.1 ± 2.3 (*p* = 0.856)	74.4 ± 44.4 (*p* = 0.680) 80.4 ± 51.6 (*p* = 0.680)
Urbanski et al. (2017) [52]	Cohort study	Adolescents: 20	5	31	Progressive adolescent and neglected adults idiopathic scoliosis	posterior rods with all screw constructs	Posterior spinal fusion only PSF w/DVR	NonDVR: 15.6 ± 1.49 DVr: 14.9 + 1.58	NR
Le Navéaux et al. (2017b) [50]	Case series	35	2	33	AIS	NR	Posterior instrumentation	16	NR
Angelliaume et al. (2017) [53]	Cohort study	70	11	59	AIS (Lenke 1 and 2)	Ti CoCr	NR	Ti (*n*:35) 16.6 ± 4 CoCr (*n*:35) 15.7 ± 2	NR
Lonner et al. (2017) [54]	Case control study	851	183	668	AIS	PJK + PJK−	Posterior approach	14.4	NR
Kim et al. (2017) [55]	Case control study	106	10	96	AIS	+ DVR simple rod derotation w/o DVR	Distal fusion, posterior approach	DVR: 15 NO DVR: 14.9	37.2 76.8
Panya-amornwat et al. (2017) [56]	Cohort study	29	5	24	AIS	Simple rod derotation (SRD) DVR using VCM (VCM)	Posterior approach w/ rod derotation Posterior approach w/direct vertebral rotation	SRD: 14.8 ± 1.7 VCM: 15.8 ± 1.8	NR
Sudo et al. (2016) [57]	Case series	64	7	57	AIS	TK < 15 TK > 15	Posterior spinal correction with fusion with segmental pedicle screw instrumentation	14.8	NR
Kokabu et al. (2016) [58]	Cohort study	49	1	48	AIS	Angle of rod deformation > 14	Posterior segmental pedicle screw instrumentation and fusion	15.5 ± 2.2	NR
Gehrchen et al. (2016) [59]	Cohort study	129	24	105	AIS	Circular rods Beam-like rods	Posterior fusion with pedicle screw	Circular rods: 15.7 ± 2.0 Beam-like rods: 16.5 ± 2.7	NR
Huang et al. (2016) [60]	Cohort study	39	9	30	AIS Lenke 5C	Simple rod derotation (SRD) Vertebral column manipulator (VCM)	Posterior pedicle screw rod Instrumentation	16.5 ± 3.3 15.8 ± 3.4	34.4 30.3
Seki et al. (2016) [61]	Case series	30	2	28	AIS	Thoracic curve (Lenke 1 and 2) Thoracolumbar or lumbar (Lenke V)	Rod reduction and differential rod contouring, followed by DVR using uniplanar screws	14.1 ± 3.1	NR
Sudo et al. (2015) [62]	Case series	21	2	19	AIS Lenke 2	NR	Posterior curve correction with SDRRT	15.8 ± 3.1	32.4
Pankowski et al. (2016) [63]	Cohort study	38	6	32	AIS	Posterior rods Pedicle screws	PLIF	15.8	NR
Liu et al. (2015) [64]	Cohort study	77	21	56	AIS	Group A: low-stiffness rod with low density of screw placement Group B: low-stiffness rod with high density of screw placement Group C: high-stiffness rod with low density of screw placement Group D: high-stiffness rod with high density of screw placement	Posterior surgery	15.79 ± 3.21	16.56 ± 6.24
Terai et al. (2015) [65]	Cohort study	52	3	49	AIS	Group N: treated with the new technique using 6.35 mm diameter different-stiffness Ti rods Group C: treated with conventional methods (correction started on the concave side) using 5.5 mm diameter Ti alloy rods	Posterior surgery	16 18.8	NR
Tang et al. (2015) [66]	Cohort study	81	6	75	AIS	DVBD: vertebral body derotation SRD: simple rod derotation	Posterior surgery Posterior surgery	14.9 ± 1.8 15.1 ± 1.6	48
Takahashi et al. (2014) [67]	Cohort study	38	0	38	AIS	Ponte group Control group	NR NR	15.6 ± 2.0 (p = 0.122) 14.4 ± 2.5 (p = 0.122)	NR
Huang et al. (2014) [68]	Cohort study	93	14	79	AIS	Cotrel–Dubousset Horizon (CDH) M10 system with a 6.35-mm rod (CDH M10 group) CDH M8 was used with a 5.5-mm rod (CDH M8 Group)	NR	15.6 ± 2.2	63.5 ± 25.5
Clément et al. (2014) [69]	Case series	99	NR	NR	AIS	Simultaneous translation on two rods (ST2R)	NR	14.8	NR
Sales et al. (2014) [70]	Other	107	NR	NR	AIS	Study 1—Mazda et al. Study 2—Jouve et al.	Anterior release Posterior approach UC used without anterior approach	15 32	NR NR
Cao et al. (2014) [71]	Meta-analysis	1615	NR	NR	AIS	Hybrid construct pedicle screw	NR	15	NR
Sudo et al. (2014) [72]	Cohort study	32	3	29	Lenke 1 thoracic AIS	Posterior rods and pedicle screws	Posterior main thoracic curve correction using SDRRT	15.0 ± 2.6	42 ± 15
Lamerain et al. (2014) [73]	Cohort study	90	20	70	AIS	CoCr rods: 64 SS rods: 26	Posterior spinal fusion and instrumentation, using all-pedicle screw constructs	15.2	30.6
Voleti et al. (2014) [74]	Case series	3	1	2	AIS	Posterior rods and pedicle screws	NR	NR	NR
Prince et al. (2014) [75]	Cohort study	352	70	281	AIS	5.5 mm rod—screw only: 73 6.35 mm rod—screw only: 12 5.5 mm rod—hybrid: 90 6.35 mm rod—hybrid: 177	Posterior	5.5 mm rod: 14.4 ± 1.8 6.35 mm rod: 14.1 ± 1.8	NR
Di et al. (2013) [76]	Case series	62	53	9	AIS	DR group Non-DR group	Posterior	NR	3.7 years
Okada et al. (2013) [77]	Cohort study	65	NR	NR	AIS patients treated using segmental pedicle screw fixation	SS: 27 (S group) Ti: 38 (T group)	Posterior correction and fusion surgery	14.4 ± 3.5 years	S GROUP: 34.7 ± 5.5 T group: 25.2 ± 2.8
Demura et al. (2013) [78]	Cohort study	26	23	3	AIS patients with thoracic curves (Lenke 1 and 2)	NR	Posterior instrumentation and fusion Ponte osteotomy	13.6 ± 1.5 years	NR
Tsirikos et al. (2012) [79]	Case series	212	24	188	AIS	Group 1 bilateral segmental pedicle screw fixation Group 2 unilateral segmental pedicle screws	Posterior Posterior	14.8 14.8	3.5 years
Anekstein et al. (2012) [80]	Case series	40	11	29	AIS patients were treated with posterior fusion using all-pedicle-screw construct with correction done through the convex side	NR	Posterior arthrodesis of the spine	15.2 years	24.9 months
Larson et al. (2012) [81]	Cohort study	28	1	27	AIS	Selective fusion Long fusion	NR	14.3 14.1	20 years
Clément et al. (2011) [82]	Cohort study	62	8	54	AIS	Simultaneous translation on two rods	PSF	14.8	44
Khakinahad et al. (2012) [83]	Case series	63	21	42	Clinical charts and radiographs of patients with AIS who were 11–19 years of age at the time of surgery and had Lenke type 1 deformity corrected by a selective thoracic fusion (lowest instrumented vertebra of T12 or L1) and had a minimum 2-year follow-up were retrospectively reviewed	Posterior spinal fusion and instrumentation	Posterior	15.8 ± 2.1	NR
Qiu et al. (2011) [18]	Cohort study	48	NR	NR	AIS	VCA technique Derotation maneuver	NR	Group A: 15.2 ± 4.8 Group B: 16.1 ± 5.5	16.8 17.5
Abul-Kasim et al. (2011) [24]	Cohort study	116	22	94	AIS	Groups according to year of operation Group 1: 2005–2006 Group 2: 2007 Group 3: 2008 Group 4: 2009	Posterior approach	15.9 ± 2.8 years	NR
Mladenov et al. (2011) [84]	Cohort study	30	NR	NR	AIS	Simple rod rotation technique (SRR) direct vertebral derotation (DVD)	Posterior only approach	14.65 (range 3.8)	32.2 (15.6)
Canavese et al. (2011) [85]	Case series	32	3	29	AIS	Posterior fusion with the multisegmented hook and screw instrumentation	Posterior	14.6 ± 1.4	72.0 ± 16.7
Clément et al. (2011) [82]	case series	24	2	22	AIS patients with hypokyphosis (T4–T12 < 20°)	AIS with hyphokyphosis	Posterior spinal fusion	14.6	49.2 (24–89)
Dalal et al. (2011) [86]	Cohort study	210	48	162	Adolescent idiopathic thoracic scoliosis	Uniplanar screw group Polyaxial screw group	Posterior spinal instrumentation	15	NR
Lamartina et al. (2011) [87]	Cohort study	36	8	28	A continuous series of 36 King 2–Lenke 1 B/C idiopathic scoliosis patients was retrospectively included in the present study Eighteen patients with a major thoracic idiopathic scoliotic curves (screw group) treated between 2006 and 2008 with screws alone and 18 similar thoracic idiopathic scoliotic curves (hybrid group) treated between 2003 and 2005 with hooks and screws were retrospectively evaluated	Screw group: all screw construct Hybrid group: pedicle screws and hooks	Posterior approach Posterior approach	19	NR
Lavelle et al. (2016) [88]	Cohort study	22	NR	NR	AIS	NR	Posterior only Cotrel–Dubousset instrumentation	35 (age at follow-up)	240
Miyanji et al. (2018) [89]	Cohort study	161	31	130	AIS	PSIF group	PSIF	PSIF: 15.3 ± 2.0	2
Li et al. (2018) [90]	Cohort study	77	9	68	AIS	Posterior selective fusion	Posterior	PSF: 14.7 ± 2.2 (p = 0.844)	PSF: 80.4 ± 15.2 (p = 0.002)
Geck et al. (2013) [91]	Cohort study	42	NR	NR	AIS	Posterior spinal fusion	PSF	NR	NR

*AIS* adolescent idiopathic scoliosis; *CDH* Cotrel–Dubousset Horizon; *CoCr* cobalt–chromium; *CTA* conventional titanium alloy; *DR* direct vertebral rotation group; *DVBD* direct vertebral body derotation; *DVD* direct vertebral derotation; *DVR* direct vertebral rotation; *HC* hybrid construct; *LIV* lowest instrumented vertebra; *MC* modified construct; *PJK* proximal junctional kyphosis; *PLIF* posterior lumbar interbody fusion; *PSF* posterior spinal fusion; *PSIF* posterior spinal instrumentation and fusion; *RCT* randomized controlled trial; *RLR* rod-link reducer; *SC* standard construct; *SDRRT* simultaneous double-rod rotation technique; *SNT* superelastic shape-memory alloy; *SRD* simple rod derotation; *SRR* simple rod rotation technique; *SS* stainless steel; *ST* simultaneous translation; *TCT* traditional corrective techniques; *Ti* titanium; *TK* thoracic kyphosis; *UC* universal clamp; *VCA* vertebral coplanar alignment; *VCM* vertebral column manipulator

### Meta-analyses

#### Impact of rod material

##### Surgical outcomes

**Fig. 2:**
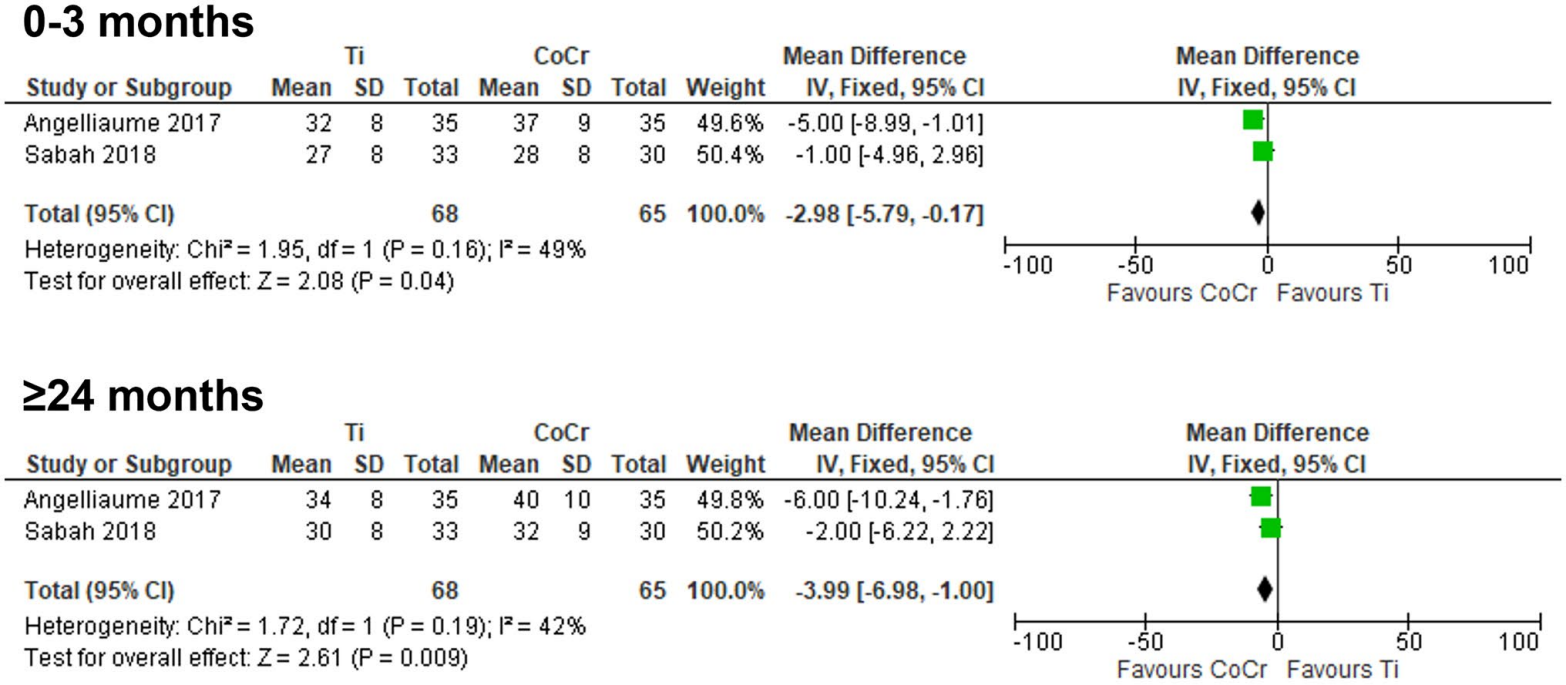
Direct comparison of kyphosis angle correction by rod material

*Kyphosis angle correction: *Two studies directly compared the use of Ti and CoCr rods and their effect on postoperative kyphosis angle correction over 0–3 months [21, 53] and ≥ 24 months [21, 53] (Fig. 2). The meta-analysis results revealed that CoCr rods provided significantly better postoperative kyphosis angle correction when compared to Ti rods, not only during a relatively shorter follow-up period (0–3 months, MD = − 2.98°, 95% CI −5.79 to − 0.17°, *p* = 0.04), but also during a relatively longer follow-up period (≥ 24 months, MD = − 3.99°, 95% CI − 6.98° to − 1.00°, *p* = 0.009).

*Coronal angle correction:* Two studies compared the use of Ti and CoCr rods and their effect on postoperative coronal angle over ≥ 24 months (Supplemental Fig. S1) [21, 53]. The overall pooled MD between the two groups was 0.50°(95% CI − 2.15° to 3.15°) and was not statistically significant (*p* = 0.71). The indirect comparative analysis evaluating coronal angle correction included seven studies evaluating Ti rods [19, 28, 64, 68, 70, 72, 77] [pooled MD 73.69% (95% CI 68.05–79.32%)], three studies evaluating stainless steel rods [77–79] [pooled MD 71.91% (95% CI 63.63–80.19%)], and three studies evaluating CoCr rods [33, 47, 49] [pooled MD 64.88% (95% CI 59.57–70.19%)]. There were not statistically significant differences in percent change in coronal Cobb angle among the varying rod materials (Chi^2^ = 5.35; *p* = 0.07; Supplemental Fig. S2).

##### Postoperative complications

*Proximal junctional kyphosis:* The two direct comparative studies also presented the data on the risk of proximal junctional kyphosis stratified by rod material (Ti rods vs*.* CoCr rods; Supplemental Fig. S3) [21, 53]. The pooled risk ratio of proximal junctional kyphosis between the two groups showed no significant difference (RR = 1.28; 95% CI 0.30–5.54; *p* = 0.74). Two studies using Ti rods reported at least one case of PJK in AIS patients undergoing posterior spine deformity surgery (Supplemental Fig. S4) [21, 53]. The overall pooled proportion for PJK was 4% (95% CI 0.0–9.0%) in patients who utilized Ti rods. Three studies which used CoCr rods reported an overall pooled proportion of 3% (95% CI 0.0–6.0%) [21, 38, 53]. In the pooled indirect comparison, the test for subgroup difference showed no significant differences between rod materials (Chi^2^ = 0.19, *p* = 0.67).

*Revision surgery:* Three studies using Ti rods reported revisions [53, 74, 82]. The overall pooled proportion for revision was 6% (95% CI 0.0–12.0%). Two studies using cobalt–chromium rods reported revision surgery with an overall pooled proportion of 4% (95% CI 0.0–8.0%) [53, 73]. One study reporting revision surgery used stainless steel rods [73]. In the pooled indirect comparison, no significant differences between rod materials were observed (Chi^2^ = 0.65, *p* = 0.72; Supplemental Fig. S5).

*Reoperation*: Four studies using CoCr rods reported reoperation in AIS patients who underwent spine deformity surgery (Supplemental Fig. S6) [38, 49, 59, 72]. The overall pooled reoperation rate was 2% (95% CI 0.0–3.0%) for CoCr rods. Only one study using stainless steel and another study using Ti rods reported reoperation rates [82]. Thus, the test for subgroup difference could not be performed due to the small number of studies.

*Infection*. Four studies using titanium rods reported postoperative infections in AIS surgery with pedicle screw fixation systems (Supplemental Fig. S7) [21, 70, 77, 79]. The overall pooled proportion of postoperative infection was 2% (95% CI: 0.0–3.0%) with titanium rods. Six studies using cobalt chromium rods reported postoperative infection with a pooled proportion of 4% (95% CI 2.0–6.0%) [20, 21, 38, 48, 49, 73], while two studies using stainless steel rods reported a pooled infection rate of 8% (95% CI 0.0–18.0%) [73, 77]. In the pooled indirect comparison, the test for subgroup difference showed no significant differences among rod materials (Chi^2^ = 4.17, *p* = 0.12).

#### Impact of rod diameter

##### Surgical outcomes

*Kyphosis angle correction*: No studies directly compared the impact of rod diameter on postoperative kyphosis angle. Three studies utilized 6 mm posterior rods for AIS surgery and reported corresponding change in the kyphosis angle [21, 22, 82]. The pooled MD in change in kyphosis angle with 6 mm rods was 13.69° (95% CI: 8.54°–18.84°). Similarly, three eligible studies utilizing 5.5 mm rods reported corresponding change in kyphosis angle were also analyzed [26, 52, 67]. Our analysis revealed a pooled MD of 10.05° (95% CI 8.53°–11.57°) in kyphosis angle. Further, when subgroups were analyzed, the test for subgroup difference showed no significant differences in kyphosis angle change between rods of 5.5 and 6 mm diameters, respectively (Chi^2^ = 1.77; *p* = 0.18) (Supplemental Fig. S8).

Coronal Angle Correction: Two studies reported on 5.5 mm and 6.35 mm rods and directly compared their effect on postoperative coronal angle at 6- to 12-month follow-up period (Supplemental Fig. S9) [60, 64]. Our analysis showed no statistically significant difference between postoperative coronal angles among the two groups (MD = 1.63, 95% CI − 0.35° to 3.61°, *p* = 0.11). Further, no significant heterogeneity was observed among the studies (*I*^2^ = 0%, *p* = 0.96). Three studies directly compared the use of 5.5 mm and 6.35 mm rods and their effect on percent change in coronal angle at follow-up period 6–12 months [60, 64, 75]. The pooled MD showed no significant difference between change in coronal angle of the two groups (MD = 2.81%; 95% CI − 5.94 to 11.57%; *p* = 0.53; Supplemental Fig. S10).

Three studies that utilized 6.35 mm rods reported percent change in the coronal Cobb angle of AIS patients who underwent spine deformity surgery with pedicle screw fixation systems (Supplemental Fig. S11) [60, 64, 75]. The pooled MD was 69.80% (95% CI 56.43–83.17%). Thirteen studies that used 5.5-mm diameter rods reported relatively higher percent change in the coronal cobb with a pooled MD of 73.01% (95% CI 69.61–76.42%) [19, 32, 35, 46, 47, 60, 64, 70, 75, 77, 78, 86]. On the other hand, three studies which used 6-mm rods reported similar percent change in the coronal cobb angle with a pooled MD of 67.65% (95% CI: 60.88–74.42%) [22, 33, 49]. The test for subgroup difference showed no significant difference in the results among varying rod diameters (Chi^2^ = 2.02, *p* = 0.36).

##### Postoperative complications

*Revision surgery*: Two studies which used 6-mm diameter rods reported having at least one case of revision surgery in AIS patients who underwent spine deformity surgery with pedicle screw fixation systems (Supplemental Fig. S12) [73, 82]. The overall pooled proportion for revision surgery was 6% (95% CI 2.0–9.0%) in patients who utilized 6-mm diameter rods. One study with at least one case of revision surgery used 6.35-mm diameter rods [74]. Test for subgroup difference was not done due to the small number of studies.

**Fig. 3:**
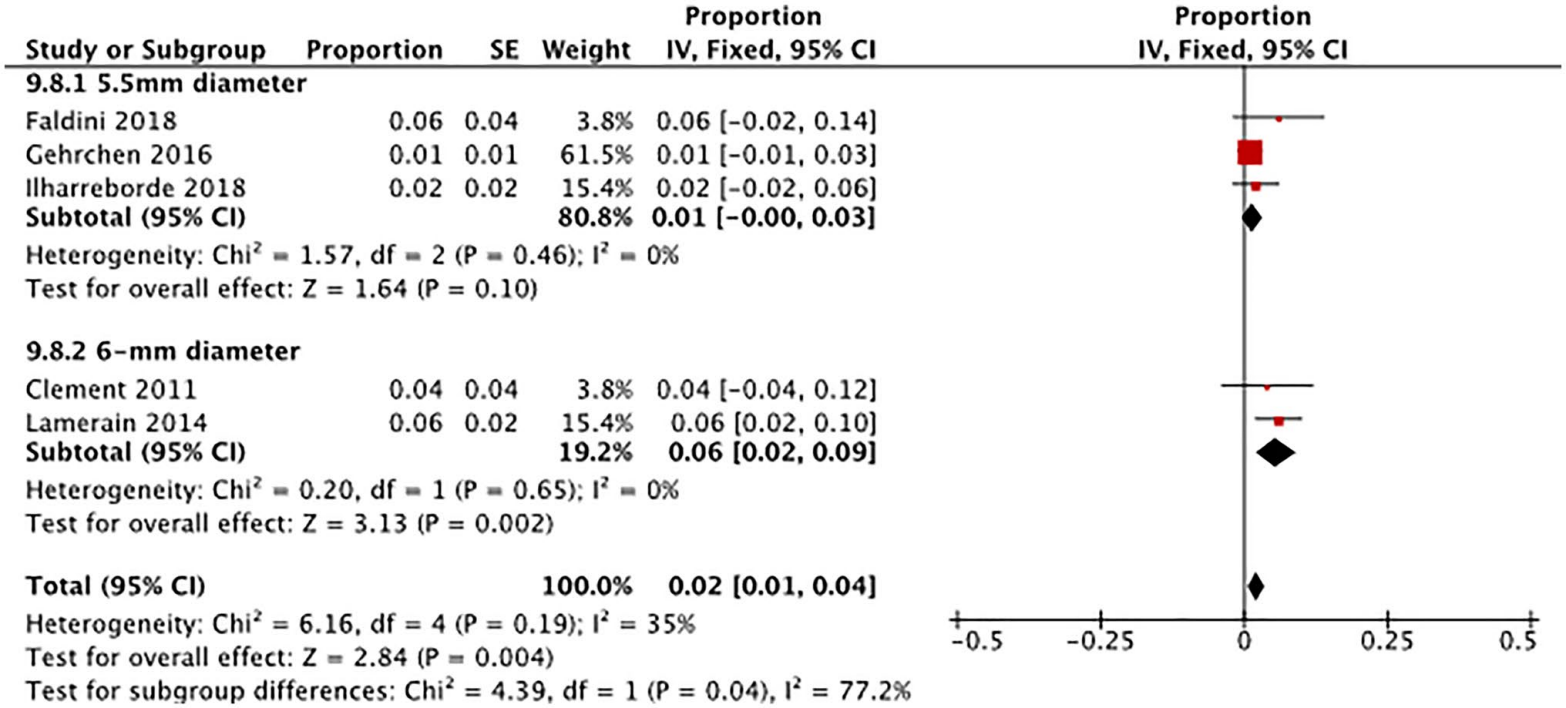
Indirect comparison of reoperation by rod diameter

*Reoperation*: Three studies utilizing 5.5 mm rods reported having at least one case of reoperation in pediatric patients who underwent spine deformity surgery (Fig. 3) [20, 38, 59]. The overall pooled proportion for reoperation surgery was 1% (95% CI 0.0–3.0%) in patients who utilized 5.5 mm diameter rods. Two studies which used 6-mm diameter rods reported having at least one case of reoperation with an overall pooled proportion of 6% (95% CI 2.0–9.0%) [73, 82]. Test for subgroup difference showed a significant difference in proportion of reoperation between the two rod diameters, with the 6 mm diameter rod having a higher propensity for reoperation (Chi^2^ = 4.39, *p* = 0.040; Fig. 3).

*Infection*: Three studies which used 6-mm diameter rods reported having at least one case of postoperative infection in AIS patients who underwent spine deformity surgery with pedicle screw fixation systems (Supplemental Fig. S13) [21, 49, 73]. The overall pooled proportion for infection was 4% (95% CI 2.0–7.0%) in patients who utilized 6-mm diameter rods. Six studies which used 5.5-mm diameter rods reported having at least one case of infection with an overall pooled proportion of 2% (95% CI 1.0–3.0%) [20, 38, 48, 70, 77, 79]. Test for subgroup difference showed no significant differences between rod diameters (Chi^2^ = 2.69, *p* = 0.10).

## Discussion

The choice of rod used for the correction of scoliosis is an important consideration in the treatment of AIS. There is substantial force exerted in AIS correction and contoured rods must be able to withstand deformation. Composition and design of the spinal rod must strike a complex balance: the rod must be flexible enough for the surgeon to bend in the desired curve, have a high enough bending yield strength that the rod maintains the bent-in-curve throughout the procedure, and have a high enough fatigue strength that it does not fracture during the therapeutic lifetime of the implant (6–24 months for a solid fusion). The rod’s ability to resist deformation or fracture brought about by contouring will depend on the material used and the diameter and shape of the rod. There have been significant changes in the types of rods and the materials used for rods over the years. Initially, Harrington rods consisted of stainless steel (SS). Present day rod constructs are more likely to consist of either Ti or CoCr.

This systematic review and meta-analysis identified 75 studies evaluating the surgical management of AIS using pedicle screw fixation systems; among which 46 studies described rod material and diameter. Study findings showed that CoCr rods provided better correction of thoracic kyphotic angle compared to Ti rods, not only during a relatively shorter follow-up period (0–3 months), but also during a relatively longer follow-up period (≥ 24 months) (*p* < 0.05). Differences in coronal angle, lumbar lordosis, proximal junctional kyphosis, revisions, and infections did not statistically significantly differ among rods of different materials or diameters. Overall, surgical treatment in patients with AIS using pedicle screw fixation systems had low complication and reoperation rates. Infections varied from 2% for patients receiving 5.5 mm rods to 4% for 6 mm rods (*p* > 0.05). Reoperation rates varied from 1% for 5.5 mm to 6% for 6-mm diameter rods and were significantly lower with 5.5 mm rods (*p* = 0.04).

There is a need for improved rod yield strength that will help maintain kyphosis and reduce intra and postoperative loss of correction [92]. Within the evolution of pediatric spinal deformity corrections, surgical technique has evolved to allow for higher degrees of derotation. As surgeons attempt these more aggressive techniques, they have begun observing an inability of the rod to maintain the kyphosis they have bent into the rod [47, 50, 92]. This “flattening” of the curve is most often observed during the high load correction maneuvers in stiff severe curves [50, 93]. There is a need for a rod material with a high yield strength to maintain the kyphosis correction that does not require a large diameter.

Biomechanical properties of spinal rods are typically differentiated by yield strength and stiffness. Generally, Ti is characterized by high yield strength but a lower stiffness, and CoCr is characterized by a very high stiffness and low yield strength [94]. However, the potential impact of rod material properties observed in the laboratory setting are not easily extrapolated to the clinical reality [94]. The clinical performance of spinal rods is susceptible to a complicated interplay of patient, surgeon, and environmental factors [95, 96]. It is possible the answer lies in other combinations of stiffness and bending yield strength. Thus, the focus of this systematic literature review and meta-analysis was to summarize published clinical evidence from studies conducted in actual human patients. Biomechanical ex vivo studies, animal studies, and cadaver studies were not included in the evaluations. Additional high-quality clinical studies comparing biomechanical differences among rod constructs are needed [94].

As expected, when pooling observational (real-world) data [97–99], the main limitation of the current study is the heterogeneity of the patient populations evaluated, the surgical techniques and technologies employed, and the definitions of outcomes used. The current study was conducted in line with recommendations available in the literature for the use of real-world evidence in meta-analyses [100]. Since *Q* was significant and *I*^2^ was > 50%, it was appropriate to use the random-effects model (REM) to calculate pooled summary estimates. The range of I^2^ values observed in the current study (0–98%) is not inconsistent with the range of those observed in other meta-analyses of observational data.

## Conclusion

CoCr rods provided better correction of AIS thoracic kyphosis compared to Ti. Surgical AIS treatment using pedicle screw fixation systems had low complication and reoperation rates. Patients with 5.5 mm rods required fewer reoperations compared to patients with 6.0/6.35 mm rods. There is a need for rod materials that provide improved rod strength and bending yield strength in a smaller profile that will help maintain kyphosis and reduce intra- and postoperative loss of kyphosis correction.

## Supplementary Information

Below is the link to the electronic supplementary material.

Supplementary file1 (DOCX 6793 KB)
