# Supplementary material for: Systematic review and meta-analysis for the impact of rod materials and sizes in the surgical treatment of adolescent idiopathic scoliosis
Source: Spine Deform. 2022 Jun 23;10(6):1245–63. doi: 10.1007/s43390-022-00537-1 (PMC9579082; doi:10.1007/s43390-022-00537-1)

# **Supplemental Figure S1. Direct comparison of percent change in coronal angle by rod material over ≥24 months**


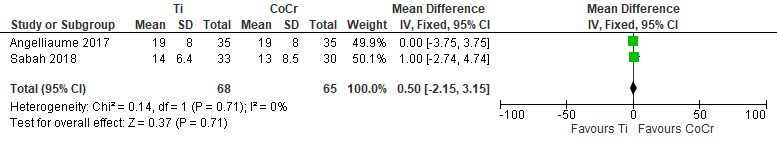


# **Supplemental Figure S2. Indirect comparison of percent change in coronal Cobb angle by rod material**


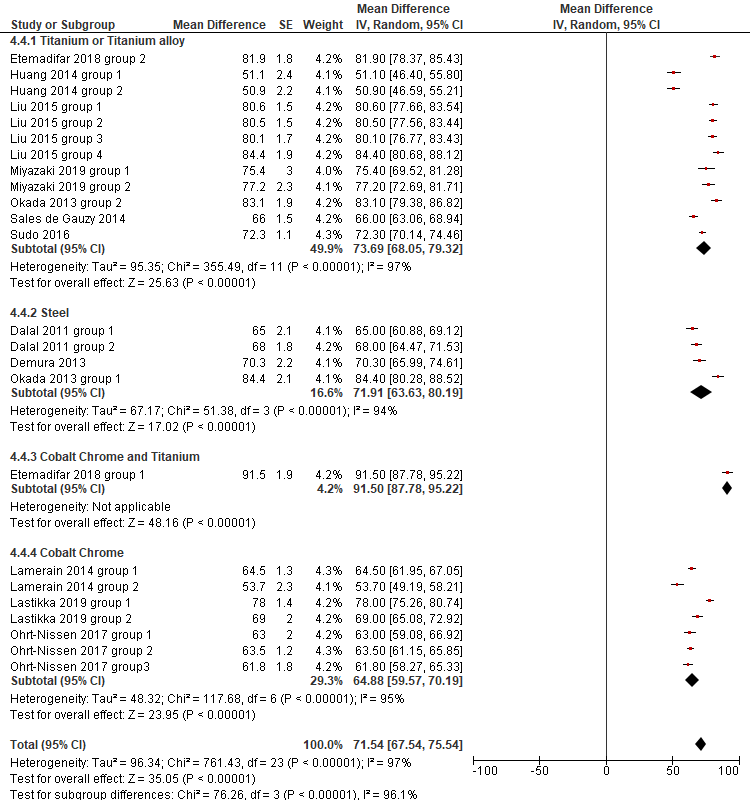


# **Supplemental Figure S3. Direct comparison of proximal junctional kyphosis by rod material**


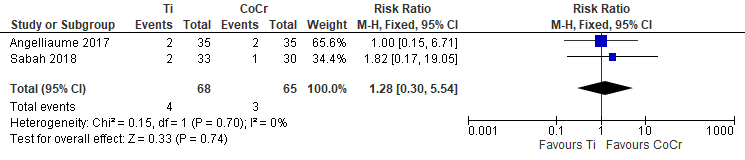


# **Supplemental Figure S4. Indirect comparison of proximal junction kyphosis by rod material**.
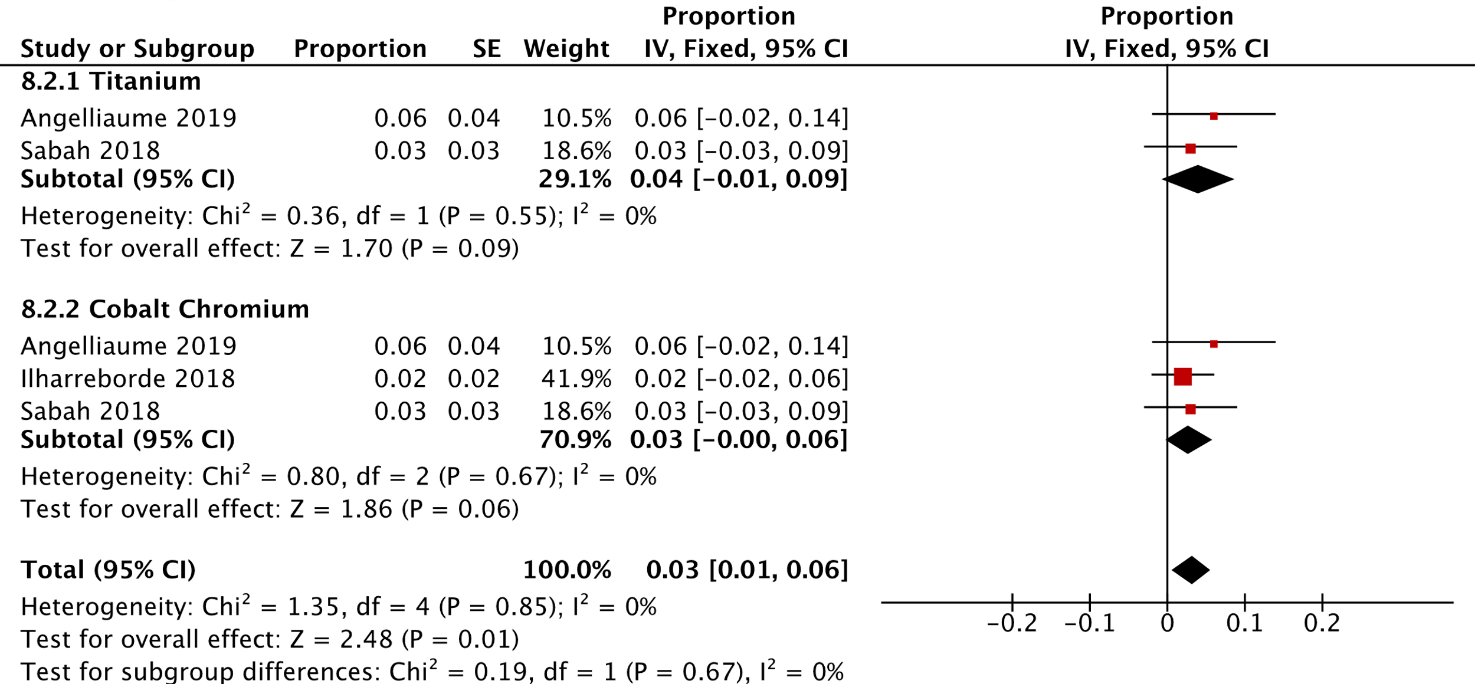


# **Supplemental Figure S5. Indirect comparison of revision surgery by rod material**


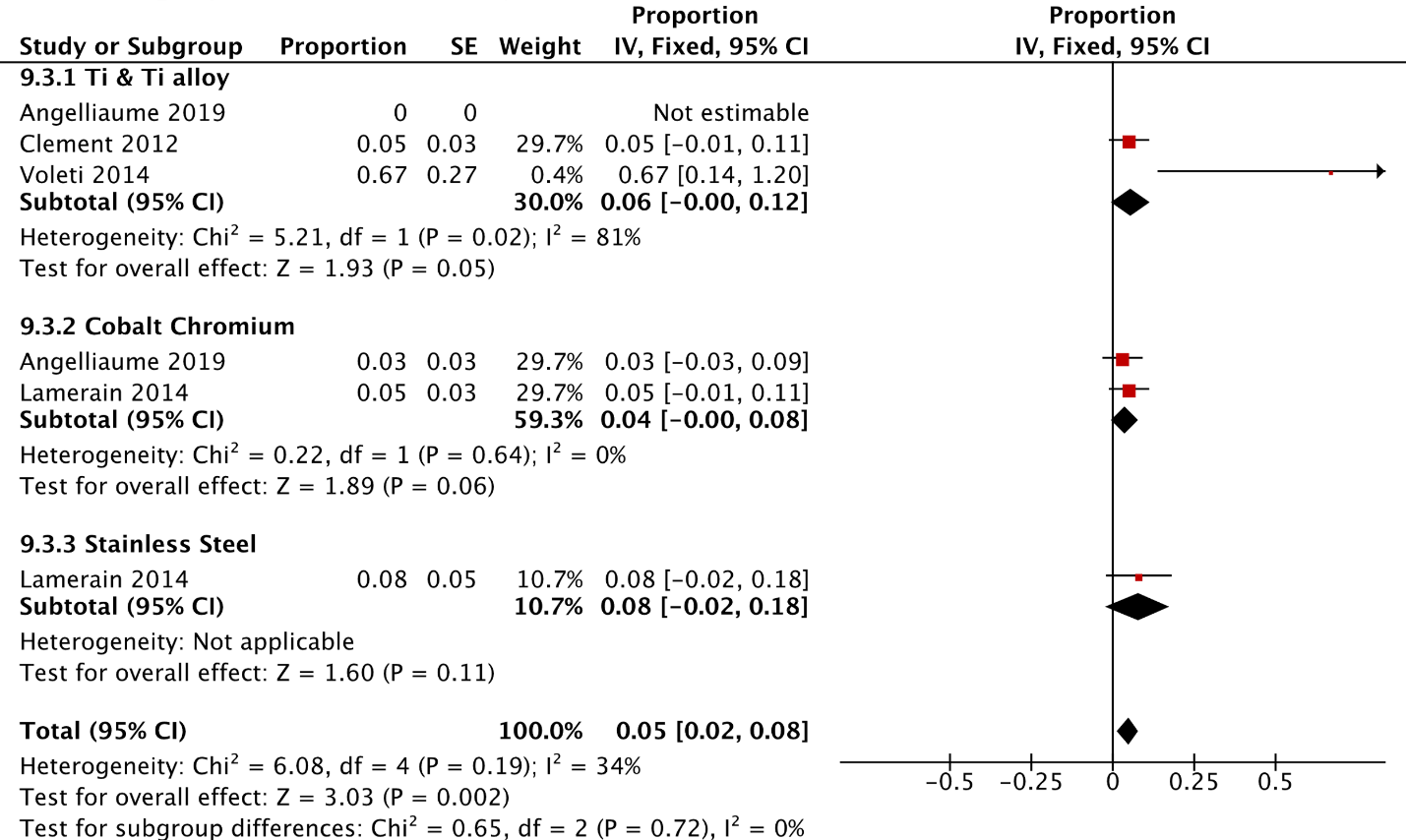


# **Supplemental Figure S6. Indirect comparison of reoperation by rod material**


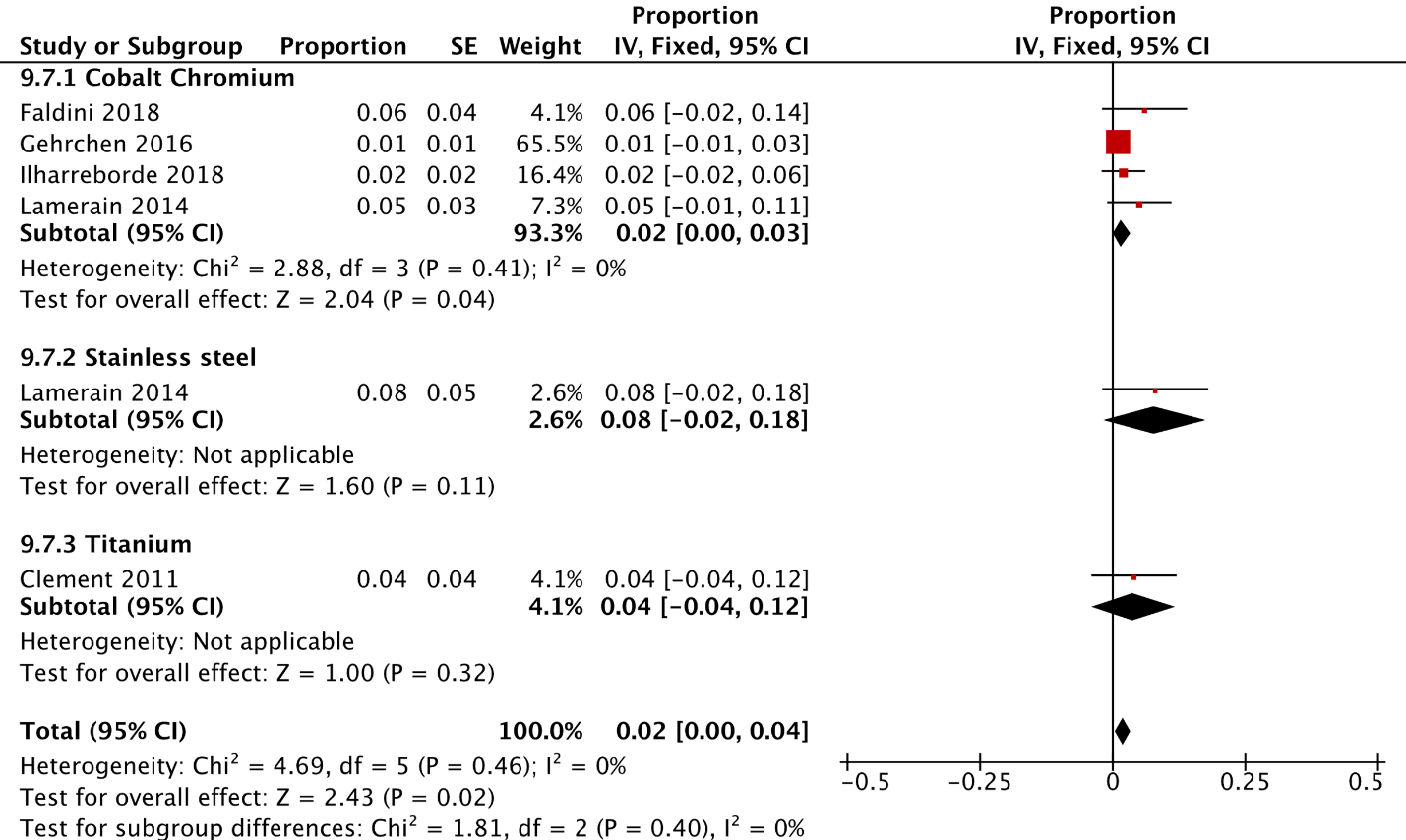


# **Supplemental Figure S7. Indirect comparison of infection by rod material**


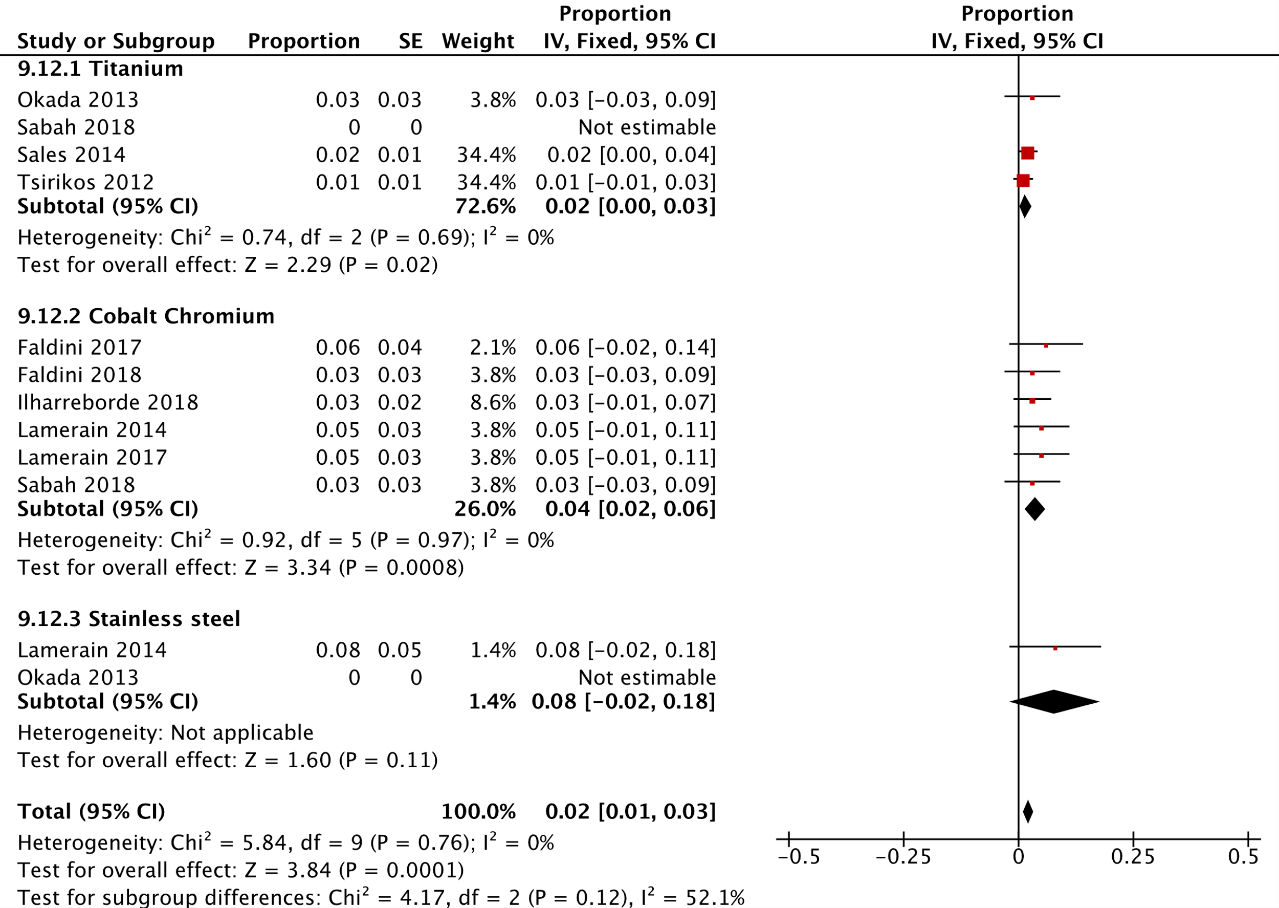


# **Supplemental Figure S8. Indirect comparison of kyphosis angle correction by rod diameter**


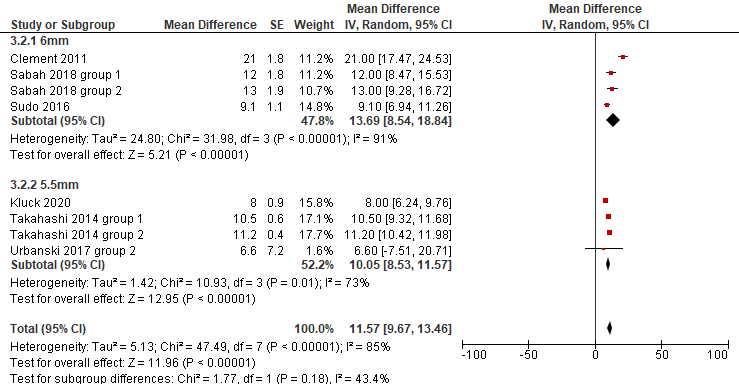


# **Supplemental Figure S9. Indirect comparison of percent change in coronal Cobb angle by rod diameter**


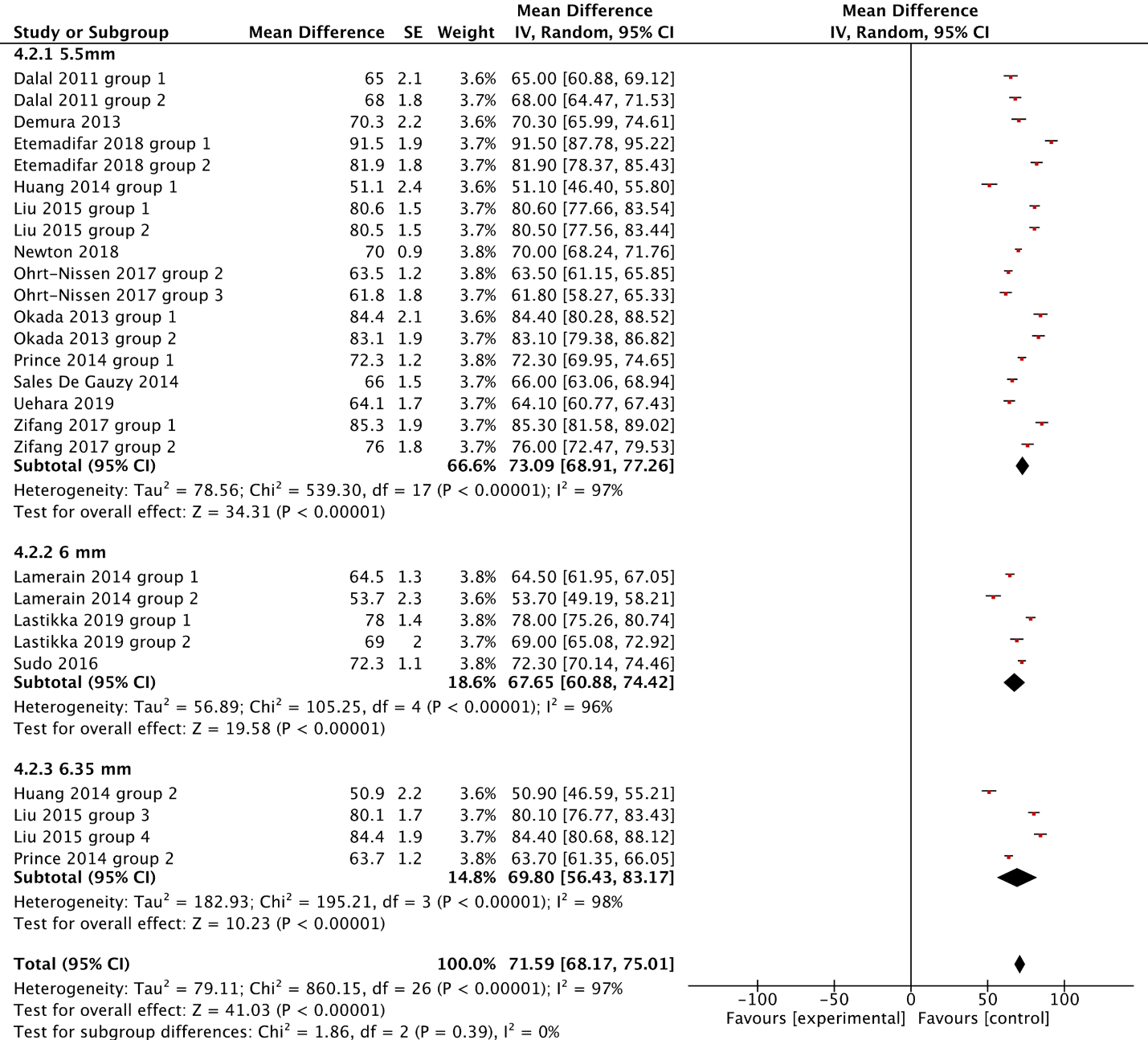


# **Supplemental Figure S10. Direct comparison of coronal angle correction by rod diameter**

≥6 to 12 months


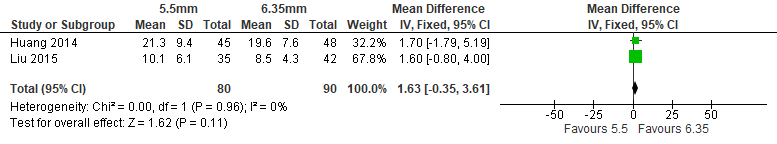


6 to 12 months


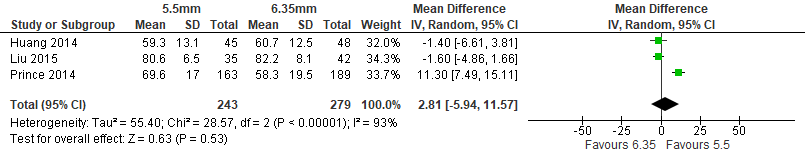


# **Supplemental Figure S11. Indirect comparison of change in lumbar lordosis by rod diameter**


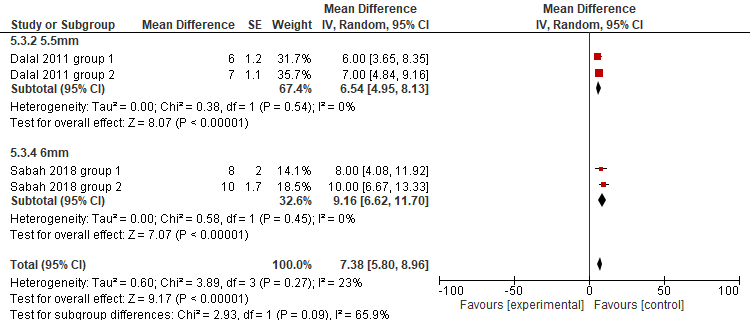


# **Supplemental Figure S12. Indirect comparison of revision surgery by rod diameter**


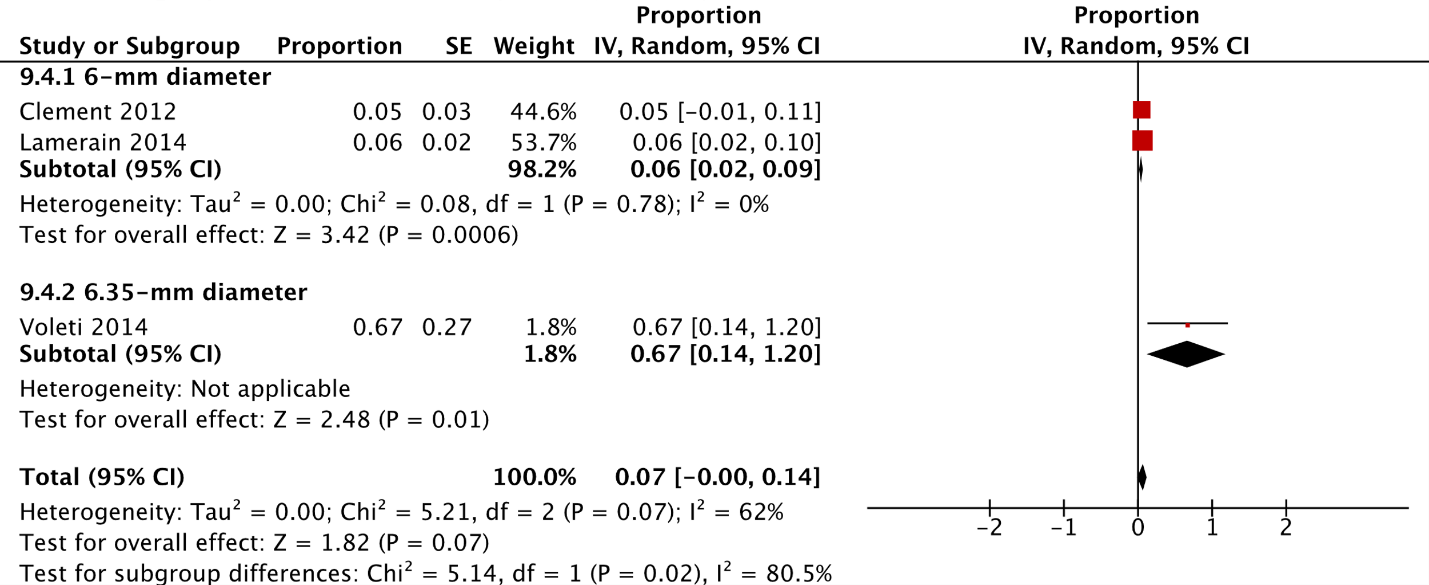


# **Supplemental Figure S13. Indirect comparison of infection by rod diameter**


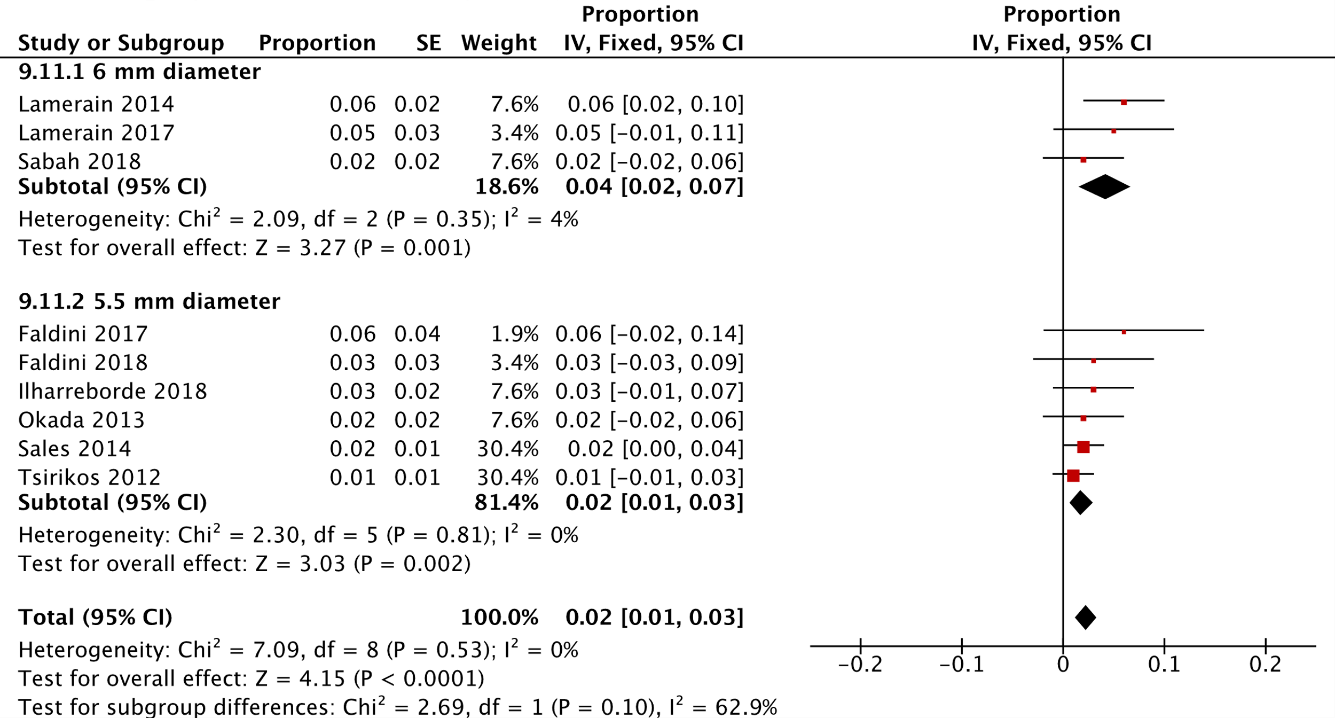

Supplement: Supplementary file 1 — Supplementary file1 (DOCX 6793 KB) [file 43390_2022_537_MOESM1_ESM.docx]
